# Thottapalayam Virus, a Prototype Shrewborne Hantavirus

**DOI:** 10.3201/eid1307.070031

**Published:** 2007-07

**Authors:** Jin-Won Song, Luck Ju Baek, Connie S. Schmaljohn, Richard Yanagihara

**Affiliations:** * Korea University College of Medicine, Seoul, South Korea; † US Army Medical Research Institute of Infectious Diseases, Fort Detrick, Frederick, Maryland, USA; ‡ University of Hawaii at Manoa, Honolulu, Hawaii, USA

**Keywords:** Hantavirus, shrews, rodents, phylogeny, synopsis

## Abstract

*Thottapalayam virus* (TPMV) has been placed in the genus *Hantavirus* of the family *Bunyaviridae* by virtue of its morphologic features and overall genetic similarities to well-characterized rodentborne hantaviruses. This virus has been isolated from the Asian house shrew (*Suncus murinus*); however, whether TPMV is naturally harbored by an insectivore host or represents spillover from a rodent reservoir host is unknown. Our analysis of published and unpublished data on the experimental host range, genetics, and molecular phylogeny of TPMV supports coevolution of TPMV with its nonrodent reservoir host. Future studies on the epizootiology of TPMV and investigations of new shrewborne hantaviruses will provide additional insights into the evolutionary origin of hantaviruses in their rodent and insectivore reservoir hosts. Such investigations may also provide clues about determinants of hantavirus pathogenicity and virulence.

## Abstract 2

This virus is antigenically and phylogenetically distinct from rodent-borne hantaviruses.

Viruses in the genus *Hantavirus*, similar to other members of the family *Bunyaviridae*, have a negative-sense, single-stranded RNA genome in 3 segments designated large (L), medium (M), and small (S), which encode an RNA-dependent RNA polymerase, envelope glycoproteins (Gn, Gc) and nucleocapsid (N) protein, respectively (*1*,*2*). Each viral genomic segment has the identical 3′-terminal sequence of AUCAUCAUCUG, which is unique to hantaviruses (*3*). However, unlike the >200 other members in this virus family, most of which have arthropod vectors, each genetically distinct hantavirus is harbored by 1 or a few closely related rodent species with which it appears to have coevolved (*4*,*5*). Hantaan virus (HTNV) shares a multimillennium relationship with the striped field mouse (*Apodemus agrarius*), Dobrava virus (DOBV) with the yellow-necked field mouse (*Apodemus flavicollis*), Seoul virus (SEOV) with the Norway rat (*Rattus norvegicus*), Thailand virus (THAIV) with the bandicoot rat (*Bandicota indica*), Puumala virus (PUUV) with the bank vole (*Myodes glareolus*, formerly *Clethrionomys glareolus*), Tula virus (TULV) with the European common vole (*Microtus arvalis*), Prospect Hill virus (PHV) with the meadow vole (*M*. *pennsylvanicus*), and Sin Nombre virus (SNV) with the deer mouse (*Peromyscus maniculatus*).

Many other rodent-hantavirus associations are known, including the recent discovery of a hantavirus in the African wood mouse (*Hylomyscus simus*) (*6*). Until recently, the 1 exception that did not have a confirmed rodent association has been Thottapalayam virus (TPMV), which was isolated from an Asian house shrew or musk shrew (*Suncus murinus*) captured in 1964 during a survey for Japanese encephalitis virus in southern India (*7*). TPMV has been classified as a hantavirus by virtue of its ultrastructural features (*8*) and overall genetic similarities with well-characterized rodentborne hantaviruses (*9*,*10*). Although isolation of TPMV predates that of all other hantaviruses, including prototype HTNV, little is known about its biology and genetics. Whether TPMV is naturally harbored by the Asian house shrew or represents recent spillover from a rodent reservoir host is unknown. We present previously unpublished data on experimental TPMV infection in small laboratory animals. We also summarize information on the antigenic and phylogenetic relationships between TPMV and rodentborne hantaviruses that may cause hemorrhagic fever with renal syndrome (HFRS) (*11*) or hantavirus pulmonary syndrome (HPS) (*12*).

## TPMV Infection in Cell Culture

Shortly after TPMV was isolated, in vitro studies involving primary cultures of guinea pig embryonic kidney, lung, and heart cells supported replication of this virus (*13*). Although mild cytopathic effect was observed in these cell cultures, the kinetics of TPMV replication was not vigorously studied. Recent adaptation of the VRC-66412 strain of TPMV to the E6 clone of Vero cells (CRL 1586) showed no cytopathic effect. At a multiplicity of infection of 0.1, intracytoplasmic, virus-specific granular fluorescence appeared somewhat later in Vero E6 cells infected with TPMV than in those cells infected with HTNV or PUUV. Strains of HTNV isolated from striped field mice and strains of SEOV from Norway rats produce large plaques (6 mm diameter) on Vero-E6 cell monolayers stained 6 days after infection with neutral red. In contrast, the VRC-66412 strain of TPMV produces much smaller plaques (≈1–1.5 mm diameter) by staining with neutral red. These plaques are easily enumerated by immunohistochemical staining 12 days after infection.

## Experimental TPMV Infection in Laboratory Animals

In their primary rodent reservoir hosts, naturally occurring and experimentally induced hantavirus infections are subclinical and chronic (*14*–*20*). Experimental infection of striped field mice and bank voles with HTNV and PUUV, respectively, is characterized by transient viremia and short-lived shedding of virus in oropharyngeal secretions; prolonged excretion of virus in urine, feces, or both; and virus persistence in tissues, particularly lung (*14*,*15*,*17*–*20*). PUUV has been serially passaged only in laboratory-bred bank voles (*17*,*19*,*20*), and strains Hällnäs and K27 of PUUV cause an asymptomatic persistent infection in Mongolian gerbils (*21*) and Syrian hamsters (*22*), respectively. Horizontal intracage transmission has been demonstrated for HTNV and PUUV, but vertical transmission does not appear to occur (*15*,*17*,*20*,*23*). In infant mice and rats experimentally infected with HTNV and SEOV, respectively, fatal meningoencephalitis develops (*24*–*27*). In contrast, mice and rats >14–21 days of age are generally resistant to experimental HTNV and SEOV infection (*26*,*27*). Conversely, infant mice are resistant to experimental infection with PUUV (*18*,*19*), PHV (L.J. Baek, unpub. data), and SNV, the prototype sigmodontine rodentborne hantavirus that causes HPS (*28*).

**Figure 1:**
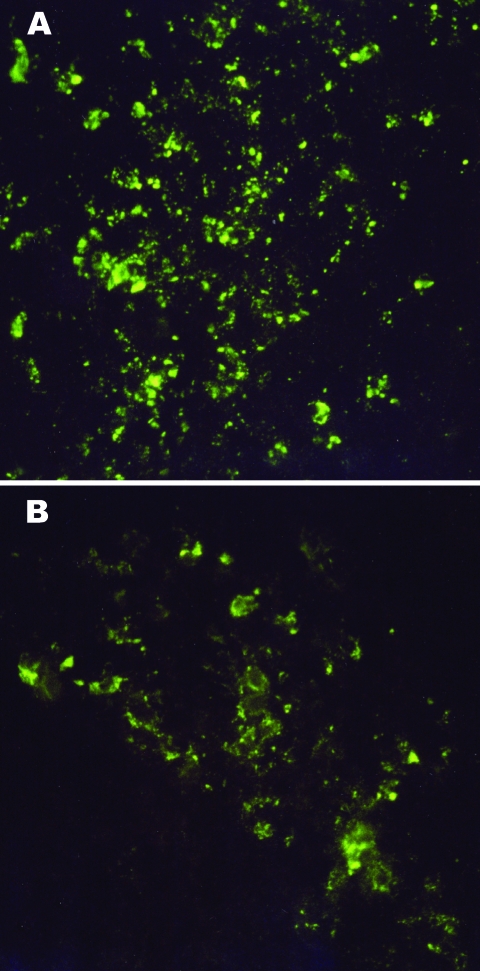
Intracytoplasmic virus-specific fluorescence in brain tissues of an 11-day-old Mongolian gerbil (A) and a 10-day-old NIH Swiss mouse (B) injected intracerebrally with 6,000 PFU of Thottapalayam virus (TPMV) strain VRC-66412 from serum of an adult rat injected intramuscularly with TPMV (original magnification, x400).

To determine the host range of experimental TPMV infection and to ascertain whether susceptibility of small laboratory animals to disseminated TPMV infection is age-dependent, we infected NIH Swiss mice and Mongolian gerbils of different ages, as well as infant deer mice and gray short-tailed opossums (*Monodelphis domestica*), by the intracerebral route with 6,000 PFU of TPMV (Table 1). Infant Swiss NIH mice, deer mice, and gerbils were equally susceptible to fatal TPMV infection. Moreover, susceptibility to disseminated TPMV infection in NIH Swiss mice and gerbils was not age-dependent, as shown by lethal meningoencephalitis (characterized by hyperexcitability, ataxia, limb paralysis, and seizures) in animals infected at 1–21 days of age. TPMV antigen was detected in cryostat-cut sections of lung, brain, kidney, spleen, and liver of experimentally infected, moribund NIH Swiss mice and gerbils (Figure 1). Thus, unlike HTNV, SEOV, PUUV, PHV, and SNV, TPMV appears to have a much broader experimental host range in small laboratory animals.

**Table 1:** Susceptibility of small laboratory animals of various ages to fatal Thottapalayam virus meningoencephalitis*

Host species	Age at injection, d	Illness onset, d	Mortality rate, %
*Mus musculus*	1	7	100
	5	7	100
	10	6	88
	14	6	94
	21	8	67
*Meriones unguiculatus*	1	9	100
	5	11	100
	11	7	80
	16	14	100
*Peromyscus maniculatus*	4	11	100
*Monodelphis domestica*	30	–	0

*Animals of various ages were injected intracerebrally with 6,000 PFU of Thottapalayam virus strain VRC-66412 and examined daily for neurologic signs and death.

Studies now in progress on experimental TPMV infection in laboratory-reared Asian house shrews should provide information about virus carriage and shedding. In addition, experimental demonstration of subclinical and chronic TPMV infection in Asian house shrews would also support the nonrodent reservoir host status.

## Antigenic, Genetic, and Phylogenetic Characterization

The antigenic relationship of TPMV with 31 other hantavirus isolates has been investigated by ELISA and cross plaque-reduction neutralization tests (PRNTs) by using antisera from experimentally infected animals (*9*). Antisera prepared against strains of HTNV, SEOV, THAIV, PUUV, and PHV have 16-fold or lower ELISA titers to cell culture-derived TPMV antigen than to homologous antigen (*9*). ELISAs with monoclonal antibodies (MAbs) prepared against HTNV showed that certain epitopes defined by Gc-specific MAbs, but not Gn-specific MAbs, are conserved among most hantaviruses, including TPMV (*9*,*29*). Similarly, cross-immunoprecipitation of radionuclide-labeled TPMV and HTNV proteins have shown conserved N and Gc glycoprotein epitopes but not Gn epitopes (*9*). Of the 32 hantaviruses examined by PRNT, TPMV is the only virus that shows no cross-neutralization with any other hantavirus, i.e., none of the heterologous antisera neutralizes TPMV, and the antiserum to TPMV does not neutralize any other hantavirus.

**Figure 2:**
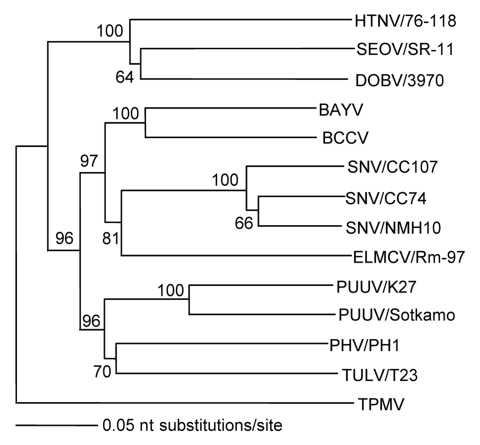
Phylogenetic relationship between Thottapalayam virus (TPMV) and other hantaviruses based on the nucleotide sequences of the full-length small (S) genomic segment, determined by using the neighbor-joining method. Numbers at each node are bootstrap probabilities (expressed as percentages) determined for 1,000 iterations. Branch lengths are proportional to number of nucleotide substitutions per site. Sequences used for comparison were those of Hantaan (HTNV/76–118, NC 005218), Seoul (SEOV/SR-11, M34881), Dobrava (DOBV/3970, L41916), Bayou (BAYV, L36929), Black Creek Canal (BCCV, L39949), Sin Nombre (SNV/CC107, L33683; SNV/CC74, L33816; and SNV/NMH10, L25784), El Moro Canyon (ELMCV/Rm-97, U11427), Puumala (PUUV/K27, L08804 and PUUV/Sotkamo, NC 005224), Prospect Hill (PHV/PH1, Z49098), and Tula (TULV/T23, Z30945) viruses. Strain designations are unavailable for BAYV and BCCV. The full-length S-segment sequence of TPMV has been deposited into GenBank (accession no. AY526097).

Apart from being antigenically distinct, TPMV also appears to be the most genetically divergent member of the *Hantavirus* genus. Full-length S-segment nucleotide and deduced amino acid sequences have nearly the same calculated distances from all other hantaviruses, which suggests an early evolutionary divergence (Table 2). Phylogenetic analysis based on the N protein–encoding S segment, as determined by the maximum parsimony and neighbor-joining methods, supports this conclusion, in that TPMV is an outgroup and all other hantaviruses segregate into clades, which parallel the evolution of murid, arvicolid, and sigmodontine rodents (Figure 2). Further elucidation of the molecular phylogeny of TPMV has been hampered by the lack of TPMV M- and L-segment sequence information. After many failed attempts, the M- and L-genomic segments of TPMV have recently been fully sequenced (J.-W. Song, R. Yanagihara, unpub. data). Full-genome analysis of TPMV shows phylogenetic relationships with rodentborne hantaviruses, which are congruent with those formed only on the basis of the S segment.

**Table 2:** Comparison of full-length small-segment nucleotide and amino acid sequences of hantaviruses with Thottapalayam virus*

Hantavirus (strain)	Thottapalayam virus	
	1,530 nt	436 aa
Hantaan (76–118)	47.9	47.1
Seoul (HR80–39)	40.8	45.7
Puumala (Sotkamo)	43.7	44.6
Prospect Hill (PH-1)	47.5	44.3
Sin Nombre (NMH10)	41.2	47.9
Andes (Chile 9717869)	44.2	47.2

*Values are percentage similarities. Distances were calculated using PAUP version 3.1.1 (Sinauer Associates Inc., Sunderland, MA, USA).

## TPMV as a Human Pathogen

Hantaviruses possess strikingly different degrees of pathogenicity for humans. Many viruses, particularly those harbored by arvicolid rodents, appear to be avirulent (such as PHV) or have low pathogenic potential (such as TULV). Among the HFRS- and HPS-causing pathogenic hantaviruses, differential use of β-3 integrins as cellular receptors on platelets and endothelial cells may account for vascular leakage and hemorrhage associated with HFRS and HPS (*30*,*31*). Preliminary studies indicate that TPMV, like PHV, uses β-1 rather than β-3 integrin (I.N. Gavrilovskaya, R. Yanagihara, unpub. data), which suggests that TPMV is nonpathogenic.

Immmunoglobulin G (IgG) against HTNV has been detected in sera from persons in southern India (*32*,*33*), but evidence for hantavirus disease in India is lacking. Nevertheless, because several species of *Apodemus* mice, including the wood mouse (*A*. *sylvaticus*), are present in India, the demonstrated seroreactivity to HTNV may represent cross-reactivity with another *Apodemus*-borne hantavirus. Alternatively, seroreactivity may indicate infection with a nonrodentborne hantavirus, such as TPMV. Because Asian house shrews are peridomestic, frequently living within or in close proximity to human dwellings, TPMV infection may occur in humans.

To begin to address this issue, researchers collected serum specimens from 363 life-long residents of Mumbai, India, during 1992 and 1993 as part of a study of retroviral infections, and tested them for serologic evidence of TPMV and SEOV infection by using the indirect immunofluorescent antibody technique. A total of 12 (3.3%) serum samples were reactive to TPMV (geometric mean titer 80.6), and 16 (4.4%) samples were reactive to SEOV (geometric mean titer 103.1). Attempts to verify the specificity of this immunoreactivity by PRNT were unsuccessful (J.-W. Song, unpub. data). More recently, however, evidence suggestive of TPMV infection was found in a Laotian immigrant with a febrile illness by using a Western immunoblot analysis and a newly developed ELISA. This ELISA used a recombinant TPMV N antigen that contained an E5/G6 epitope captured by MAb E5/G6 (*29*).

## Future Research Directions and Perspective

Although the detection of viruses in insectivores has been largely incidental or accidental, demonstration of Borna disease virus in brain tissues of the bicolored white-toothed shrew (*Crocidura leucodon*) (*34*) suggests that insectivores may play a greater role in the ecology of zoonotic diseases than previously appreciated. The prototype shrewborne hantavirus, TPMV, must be viewed within this context. Although limited data do not indicate that TPMV is a human pathogen, other shrewborne hantaviruses may be pathogenic for humans. In this regard, no one had the prescience to predict that hantaviruses harbored by sigmodontine rodents would be etiologically associated with an acute, rapidly progressive, frequently fatal respiratory illness in the Americas, now known as HPS. The realization that rodentborne hantaviruses are capable of causing diseases as clinically disparate as HFRS and HPS increases the possibility that hantaviruses harbored by nonrodent hosts may similarly cause a wide spectrum of febrile diseases or be linked with a syndrome currently of unknown etiology. Development of reagents directed toward insectivore serum proteins would greatly increase the sensitivity and specificity of serologic assays to ascertain antihantaviral immunologic responses in shrews and result in improved screening for new shrewborne hantaviruses.

Even in the absence of such reagents, several insectivore species are already known to be prime candidates for intensive investigations aimed at identifying new hantaviruses and exploring their disease associations. As examples, hantavirus antigens have been previously detected in tissues of the Eurasian common shrew (*Sorex araneus*), Eurasian water shrew (*Neomys fodiens*), and common mole (*Talpa europea*) in the former Soviet Union (*35*,*36*), and seroreactivity suggestive of hantavirus infection was found in short-tailed shrew (*Blarina brevicauda*) in the United States (*37*). In addition, hantaviruses isolated more than 2 decades ago from the greater white-toothed shrew (*Crocidura russula*) (*38*) and Chinese mole shrew (*Anourosorex squamipes*) (*38*) in Sichuan Province, People’s Republic of China, have been inadequately characterized. The probability is high that >1 of these shrewborne hantaviruses may be phylogenetically distinct.

**Figure 3:**
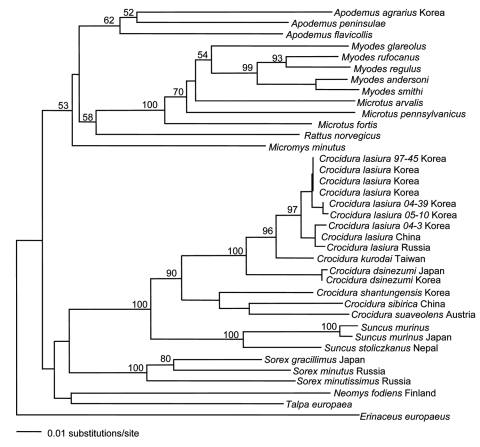
Phylogenetic relationship between *Suncus murinus* and other insectivores and rodents in a 401-nt cytochrome b region of mitochondrial DNA determined by using the neighbor-joining method. Numbers at each node are bootstrap probabilities determined for 1,000 iterations. Members of the genus *Crocidura* (white-toothed shrews) belong to the subfamily *Crocidurinae*. They are distinguished from members of the subfamily *Soricinae* (red-toothed shrews) by their unpigmented teeth, 3 upper unicuspids, and more prominent ears than either the genera *Sorex* or *Neomys*.

Another approach to the targeted discovery of new hantaviruses harbored by shrews relies on molecular phylogeny. By constructing phylogenetic trees based on mitochondrial or nuclear gene DNA sequences, existence of hantaviruses in the Korean field mouse (*A*. *peninsulae*) (*39*) and the royal vole (*Myodes regulus,* formerly *Eothenomys regulus*) (J.-W. Song, unpub. data) was correctly predicted. When this predictive paradigm is applied to insectivores, species of *Crocidura* and *Sorex* genera would be expected to serve as reservoir hosts of hantaviruses because of their close phylogenetic proximity to *S*. *murinus* (Figure 3). The recent detection of a novel hantavirus in the Therese shrew (*Crocidura theresae*) in Guinea (*40*) supports this conjecture. In addition, aided by primers based on the complete genome of TPMV, new hantaviruses have been found in 4 shrew species in the family *Soricidae* from Eurasia and the Americas (J.-W. Song, R. Yanagihara, unpub. data, and S. Arai, R. Yanagihara, unpub. data). These newly identified shrewborne hantaviruses provide new knowledge about the genetic diversity of hantaviruses as well as possible insights into their evolutionary origin through host-switching events.

Fundamental to the discovery and characterization of new hantaviruses, whether harbored by insectivores or rodents, is their relevance to human health. Because insectivore populations are generally much smaller than rodent populations, the probability of contact between humans and most insectivore species (and their excretions) may be too low for virus transmission. However, this probability is true for most zoonotic microbes, which only rarely infect humans. Thus, in the absence of disease outbreaks, zoonotic diseases frequently go unrecognized. In this regard, HPS would have similarly gone undetected had cases not clustered in time and space and had a closely knit group of dedicated and astute healthcare workers not recognized that something unusual was happening. The long-awaited clue of finding IgG against TPMV in a febrile Laotian immigrant (*29*) might indicate cross-reactivity to a pathogenic shrewborne hantavirus in Southeast Asia.
